# A Novel Long Short-Term Memory Based Optimal Strategy for Bio-Inspired Material Design

**DOI:** 10.3390/nano11061389

**Published:** 2021-05-25

**Authors:** Bin Ding, Dong Li, Yuli Chen

**Affiliations:** 1 Institute of Solid Mechanics, School of Aeronautic Science and Engineering, Beihang University, Beijing 100191, China; yulichen@buaa.edu.cn; 2 School of Engineering, Brown University, Providence, RI 02912, USA; dong_li1@brown.edu

**Keywords:** staggered structure, simultaneous superior stiffness and toughness, optimal design, long short-term memory

## Abstract

Biological materials have attracted a lot of attention due to their simultaneous superior stiffness and toughness, which are conventionally attributed to their staggered structure (also known as brick and mortar) at the most elementary nanoscale level and self-similar hierarchy at the overall level. Numerous theoretical, numerical, and experimental studies have been conducted to determine the mechanism behind the load-bearing capacity of the staggered structure, while few studies focus on whether the staggered structure is globally optimal in the entire design space at the nanoscale level. Here, from the view of structural optimization, we develop a novel long short-term memory (LSTM) based iterative strategy for optimal design to demonstrate the simultaneous best stiffness and toughness of the staggered structure. Our strategy is capable of both rapid discovery and high accuracy based on less than 10% of the entire design space. Besides, our strategy could obtain and maintain all of the best sample configurations during iterations, which can hardly be done by the convolutional neural network (CNN)-based optimal strategy. Moreover, we discuss the possible future material design based on the failure point of the staggered structure. The LSTM-based optimal design strategy is general and universal, and it may be employed in many other mechanical and material design fields with the premise of conservation of mass and multiple optimal sample configurations.

## 1. Introduction

In the material design field, it is hard to pursue high stiffness and high toughness simultaneously due to their mutual exclusiveness [1,2]. However, biological materials like nacre, bone, and tendon have provided us very good alternatives for obtaining both high stiffness and high toughness, which are the multiscale and staggered stiff mineral platelets with self-similar hierarchy embedded in the soft protein matrix at the most fundamental nanoscale level [3]. Bio-inspired composite material designs and fabrications have sprung up, such as bulk artificial nacre with bottom-up strategy [4], nacre-like alumina with rapid pressureless infiltration of the stiff Zr-based bulk metallic glass (BMG) [5], nacre-inspired structures with electrically assisted three-dimensional (3D) printing [6], and bio-inspired laminated transparent glass with a 3D synthetic technology [7]. All of the experimental attempts have validated the superior mechanical properties of the staggered structure. Besides, under the default assumption that the staggered structure is globally optimal in the aspect of simultaneous superior stiffness and toughness, many rigorous theoretical analyses have been conducted to interpret its unique tension–shear chain deformation behavior and mechanism [8,9,10,11,12,13], which can basically be described as mineral platelets under tension and protein matrix under shear. However, few studies have mentioned the fundamental baseline question of whether the staggered structure is globally optimal in the aspect of simultaneous superior stiffness and toughness at the nanoscale level. Guo et al. [14] proposed a genetic algorithm (GA)-based optimization framework to reproduce the staggered structure with simultaneously optimized stiffness and toughness, while GA is sensitive to the selection of the initial population.

Though the concept of “machine learning” (ML) was first proposed by Arthur Lee Samuel (a pioneer at IBM) in the 1950s [15], this field has not become widely popular until recently, which is mainly attributed to three reasons: matured theories, abundant data, and powerful computational capabilities. With the rapid development, ML can be applied to not only almost everywhere in our daily life, but also mechanical fields where the physical logic is clear but currently cannot be expressed in an analytical way [16,17,18,19]. Moreover, in mechanical fields, an exquisitely designed ML model can replace the ambiguous physical logic as an evaluation function for optimal design. For example, in the case of searching for the optimal design (high toughness and strength) of a two-dimensional (2D) composite structure with a pre-crack and composition of hard and soft blocks, Grace et al. [20,21] utilized the weights from the linear regression ML model to determine a better unit cell configuration at first and used the distributions of three kinds of unit cells among the top 100 samples from the previous training data to further train a better ML model and then to iteratively obtain the distributions in new top 100 samples. This optimal strategy performed well in the scenario with only one optimal distribution pattern, where soft blocks always tend to stick around the crack tip to resist the crack propagation. However, what if the problem cannot be described by linear regression model or the design space has several distribution patterns to achieve optimality? Diab et al. [22] extended the above work by implementing the GA to conduct the optimization. However, during the optimization, the crossover and mutation process changed the material ratio, so a fixed volume fraction of soft (hard) blocks could not be maintained, which led to their system not being mass-conserved and results being unconvincing. In another scenario of optimizing 2D graphene kirigami configuration for the maximum failure strain, Paul et al. [23] used a CNN model from the last iteration to loop over the whole design space to search for the CNN-predicted top 100 samples and then added them to the input dataset to further train a more accurate model. Because of symmetry, there should be at least two sample configurations to achieve optimum at the same time. However, their CNN-based optimal strategy could obtain only one of the best sample configurations, while the other one was not even in the top 5, which leaves us with the question of whether we could design an optimum strategy to obtain all of the best sample configurations at the same time.

Fortunately, the recurrent neural network (RNN) [24] provides an alternative. Compared with CNN, which is favored in computer vision, RNN is specialized in dealing with time sequences like natural language processing. By analyzing the above-mentioned optimal design problems in mechanical fields, one can see that what we care more about is the specific property, which is equivalent to the property under fixed mass (hard-to-soft material ratio). Then, the entire design space in mechanical fields actually can be transformed into a sequence with fixed numbers or letters, which can represent different distributions and different specific properties with the same material ratio. Compared with CNN, it seems that RNN may be a better tool to solve the optimal design problem under fixed mass.

**Figure 1:**
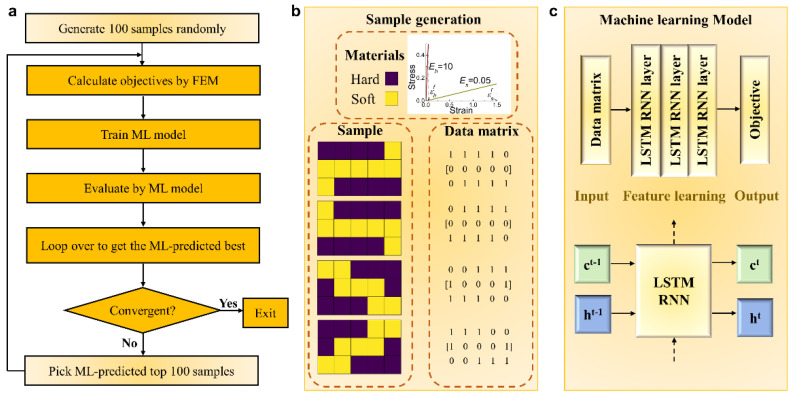
System schematic of the LSTM-based optimal strategy. (**a**) Flow chart. (**b**) Sample generation. The sample is composed of two kinds of materials with different linear elastic mechanical properties: hard material colored by purple (*E*_h_ = 10.0 GPa, *ε*^f^_h_ = 0.05) and soft material colored by yellow (*E*_s_ = 0.1 GPa, *ε*^f^_s_ = 1.5). The presented samples are the FEM-calculated top 4 sample configurations, which belong to the calculation domain discretized into 15 blocks and the material ratio of hard to soft fixed as 8/15. With blocks occupied by hard material and soft material expressed as 1 and 0 respectively, a data matrix composed of 0 and 1 can be adopted to represent each sample. (**c**) The construction of long short-term memory based machine learning model. Data matrixes composed of 0 and 1 that depict the distribution of hard and soft materials are input features. The hidden layers are followed by two LSTM layers for feature learning and one LSTM layer for regression. The objective (single value) is the output property. Each LSTM RNN layer possesses two states, which are cell state and hidden state.

In this work, in order to investigate the most fundamental baseline question of whether the staggered structure possesses simultaneous optimal stiffness and toughness at the most elementary nanoscale level, we propose a novel long short-term memory (LSTM)-based optimal strategy. LSTM [25] is one kind of RNN with both feedforward and feedback connections, which enables the applications to time-related data sequences. Figure 1a shows the flow chart of the proposed LSTM-based optimal strategy. We first randomly generate 100 samples and calculate their objectives by finite element method (FEM) as the ground truth, and then use them to train a rough ML model, which is used next as the evaluation function to predict the objectives of all possibilities in the entire design space. Note that the chosen sample number for each iteration (i.e., 100) is consistent with previous work [14,21,23]. Then, we can obtain ML-predicted top 100 samples, the ground truth objectives of which are recalculated by FEM. After that, the ML-predicted top 100 samples with the FEM-calculated objectives are added to the input dataset to update the weights of the rough ML model so as to obtain an improved ML model. These iterations come to an end when the ML-predicted best sample configurations converge to the FEM-calculated best sample configurations. Figure 1b illustrates the method of sample generation. Our calculation domain is composed of two kinds of materials (hard and soft), and there are two sample configurations with globally optimal objective (see Equation (3) for the objective). The FEM-calculated top four sample configurations and their corresponding data matrixes are presented. Similar to the fixed hard-to-soft material ratio for all the possible sample configurations in the entire design space, the proportion of “1” and “0” in any data matrix is the same, and only the order matters. Figure 1c shows the architecture of the ML model. The RNN with two LSTM layers for feature learning and one LSTM layer for regression is adopted as the ML model. Details of FEM calculation and ML model are given in Section 2. Our LSTM-based optimal strategy can provide an elegant way to solve the aforementioned optimal design with multiple optimal distribution patterns.

## 2. Methods

### 2.1. Sample Generation

**Figure 2:**
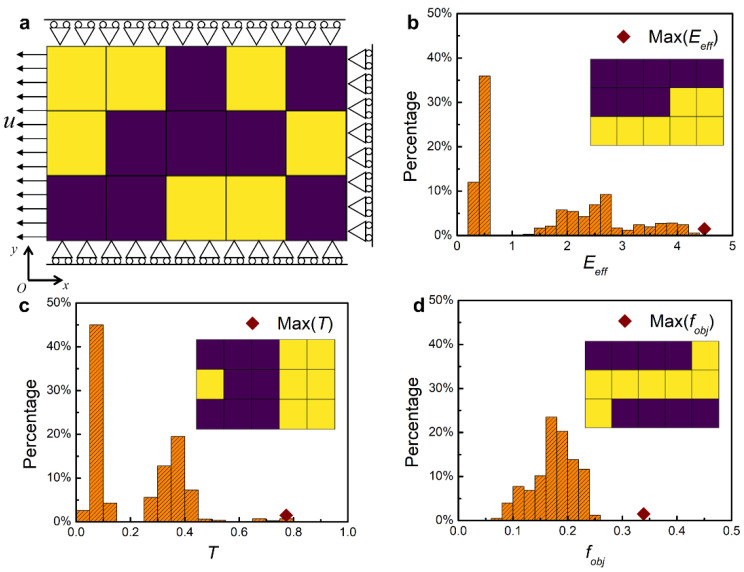
FEM calculations. (**a**) The calculation domain with quarter symmetry and under uniaxial tension. Symmetric boundary conditions are applied to the upper, bottom, and right edges, while the left edge is subject to a uniform tensile displacement loading. Here, the calculation domain is discretized into 5 × 3 = 15 blocks which are occupied by either hard material (purple) or soft material (yellow). The volume fraction of hard material is fixed at 8/15. (**b**–**d**) The FEM-calculated results of the distribution of (**b**) effective stiffness *E_eff_*, (**c**) toughness *T*, and (**d**) objective *f_obj_*. The insets in (**b**–**d**) represent the sample configurations corresponding to the maximum *E_eff_*, *T* and *f_obj_*, respectively.

The hierarchical biomaterials are typically anisotropic with load-bearing property in one direction, owing to the established fact that staggered hard plates in a soft matrix are thought to be optimal as those biomaterials evolved in nature, and then the highly efficient load transfers through a tension–shear chain mechanism [8]. Accordingly, we consider the calculation domain as a 2D representative unit cell with quarter symmetry under uniaxial tension, as shown in Figure 2a [14]. Symmetric boundary conditions are applied to the upper, bottom, and right edges, while the left edge is subject to a uniform tensile displacement loading [14]. The aspect ratio of this calculation domain is set to be 0.6 in order to capably accommodate the tension–shear chain configurations. The unit cell is discretized into a number of blocks occupied by hard or soft material. Then sample configurations can be determined by the distribution of hard and soft materials. To better illustrate the problem, we discretize the unit cell into 5 × 3 = 15 blocks here and further verify the generality and universality of our LSTM-based optimal strategy by enlarging the block number to 21 (7 × 3). With blocks occupied by hard material and soft material expressed as 1 and 0, respectively, a data matrix composed of 0 and 1 can be adopted to represent each material distribution, as shown in Figure 1b.

Considering the huge difference of deformation behavior between the hard inorganic phase and soft organic phase in nature, in this problem, we carefully set the mechanical properties as Young’s modulus *E*_h_ = 10.0 GPa and failure strain *ε*^f^_h_ = 0.05 for hard material and Young’s modulus *E*_s_ = 0.1 GPa and failure strain *ε*^f^_s_ = 1.5 for soft material (See Figure 1b for the constitutive law). The Poisson’s ratio is the same for hard and soft material: *ν*_h_ = *ν*_s_ = 1/3. Finite element method (FEM) is adopted to solve this problem, with the failure criterion chosen as the maximum principal strain criterion. The failure criterion is applicable to each block in the calculation domain regardless of whether it is occupied by hard material or soft material. Therefore, the failure criterion for the overall calculation domain can be expressed as $\max\left[{\left(\varepsilon_{h}^{p}\right)}_{i}\right]=\varepsilon_{h}^{f}\;\left(i=1,\;\ldots ,n_{h}\right)\;\mathrm{or}\;\max\left[{\left(\varepsilon_{s}^{p}\right)}_{j}\right]=\varepsilon_{s}^{f}\;\left(j=1,\;\ldots ,n_{s}\right)$, where *ε*^p^ is the maximum principal strain of an element and *n*_h_ and *n*_s_ are the numbers of hard and soft elements, respectively. Then the effective stiffness *E_eff_* along the loading direction and structure toughness *T* of the calculation domain can be expressed as

(1) $$E_{eff}=\frac{\frac{1}{n}\sum_{i=1}^{n}\sigma_{1i}^{f}}{\frac{1}{n}\sum_{i=1}^{n}\varepsilon_{1i}^{f}}\;,$$

(2) $$T=\sum_{i=1}^{n}\sum_{j=1}^{4}\frac{1}{2}\sigma_{ji}^{f}\varepsilon_{ji}^{f}\;,$$

where $n=n_{h}+n_{s}$ denotes the total number of the elements in the design domain, $\sigma_{ji}^{f}\;$ and $\varepsilon_{ji}^{f}\;$ are the *j*-th stress and strain components (with 1=xx, 2=xy, 3=yx, and 4=yy) of the element *i* in the plane-strain state when the calculation domain reaches failure. The stress and strain fields are solved by using four-node bilinear elements in FEM. To simultaneously optimize stiffness and toughness, the objective function is defined as

(3) $$f_{obj}=E_{eff}\cdot T\;,$$

In this way, a larger objective means a better configuration in the aspect of both stiffness and structure toughness. The optimization is conducted under the hypothesis of mass conservation (fixed 8 and 12 blocks occupied by the hard material in the calculation domain discretized into 15 and 21 blocks, respectively). Then the whole design spaces for 15, 21 blocks are $C_{15}^{8}\left(\sim 10^{4}\right),\;C_{21}^{12}\left(\sim 10^{6}\right)$, respectively. In the unit cell discretized into 15 blocks, we calculated all possible sample configurations in the whole design space to check the distributions of stiffness, toughness, and objective (Figure 2b–d). The insets in Figure 2b–d represent the sample configurations corresponding to the maximum *E_eff_*, *T*, and *f_obj_*, respectively. One can see that hard and soft materials tend to lie in parallel and in series along the loading direction to pursue high stiffness or high toughness. Only when stiffness and toughness are optimized at the same time can the staggered configuration be obtained. Overall, we have prepared data matrixes composed of 0 and 1 as input features and a single objective value as the output property for the ML model.

### 2.2. Recurrent Neural Network

Long short-term memory recurrent neural network was adopted to train our well-prepared data via Keras framework [26] due to the similarity between our input features and the letters in a sentence. The meaning of a sentence can be antipodal if the order of the letters changes, and the same relationship holds for our input features and the objective. As illustrated in the sample generation section, any sample configuration in the design space can be mapped into a data matrix composed of 0 and 1, which is considered as the input feature. To thoroughly show the structural arrangement and properly enlarge the gap between sample configurations with high and low objectives, the data matrix is further replicated by the symmetry rule to obtain a larger matrix with the size of 15 × 24 as the ML model input, while the objective calculated by Equation (3) is normalized as $e^{f_{obj}}/e^{\max[f_{obj}]}$ as the ML model output. Figure 1c shows the construction of the RNN, which consists of two LSTM layers for feature learning and one LSTM layer for regression with one neuron. The two LSTM layers for feature learning are determined by the neuron number of 16, dropout ratio of 0.2, and ReLU activation function. Hence, the construction of the ML model embed into our optimal strategy is confirmed. For ML model training in each iteration, the total dataset (100 data for the first iteration, ~200 data for the second iteration, ~300 data for the third iteration…) is randomly split to 70% for training and 30% for testing. To fully evaluate the iterated ML model in the aspect of both the error and the accuracy, four kinds of indicators are utilized, which are the mean square error (MSE), the mean absolute error (MAE), the explained variance score (EVS), and the coefficient of determination (i.e., R^2^):

(4) $$\mathrm{MSE}\left(y,\hat{y}\right)=\frac{1}{n}\sum_{i=1}^{n}{\left(y_{i}-{\hat{y}}_{i}\right)}^{2}\;,$$

(5) $$\mathrm{MAE}\left(y,\hat{y}\right)=\frac{1}{n}\sum_{i=1}^{n}\left|y_{i}-{\hat{y}}_{i}\right|\;,$$

(6) $$\mathrm{EVS}\left(y,\hat{y}\right)=1-\frac{Var\left\{y-\hat{y}\right\}}{Var\left\{y\right\}}\;,$$

(7) $$R^{2}\left(y,\hat{y}\right)=1-\frac{\sum_{i=1}^{n}{\left(y_{i}-{\hat{y}}_{i}\right)}^{2}}{\sum_{i=1}^{n}{\left(y_{i}-\frac{1}{n}\sum_{i=1}^{n}y_{i}\right)}^{2}}\;,$$

where y and $\hat{y}\;$ denote the FEM-calculated and ML-predicted outputs, respectively, and n is the number of samples in the considered data set in the current iteration. MSE and MAE are widely used to depict the model error with the minimum value being 0, while EVS and R^2^ depict the model accuracy with the best possible score being 1.0.

## 3. Results

**Figure 3:**
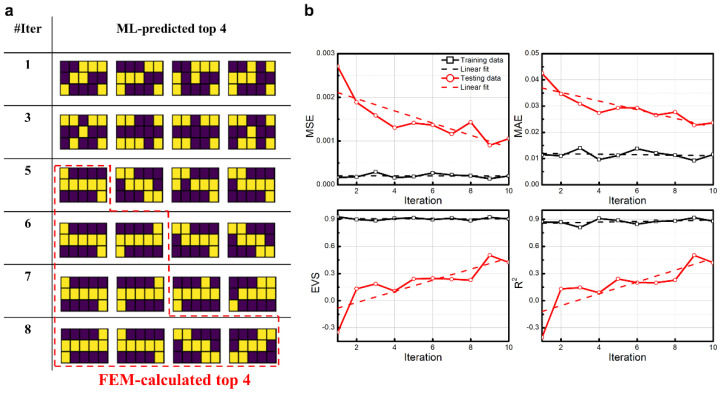
Iterated results and evaluations for the calculation domain discretized into 15 blocks by the proposed LSTM-based optimal strategy. (**a**) Top 4 ML-predicted sample configurations in each iteration. The sample configurations inside the red dashed-lined domain are the FEM-calculated top 4 sample configurations. (**b**) The relationship between evaluation indictors MSE, MAE, EVS, and R^2^ and iteration numbers. Black curves with open rectangles are for the training dataset; black dashed lines mark average values between iterations 7 and 10. Red curves with open circles are for the testing dataset; red dashed lines mark linear fit of red curves between iterations 1 and 10.

The LSTM-based optimal strategy was first applied to the calculation domain discretized into 15 blocks. Figure 3 shows the results and the performance of the iterated ML model in each iteration. The FEM-calculated top four sample configurations are presented in Figure 1b as the benchmark. The top two sample configurations share the same best objective due to the symmetry. From the ML-predicted top four sample configurations in each iteration as plotted in Figure 3a, one can see that within five iterations, only ~500 samples calculated by FEM, which account for merely 7% of the entire design space, can accurately predict the best sample configuration. In the sixth iteration, the ML model can obtain both of the two best sample configurations (staggered structure). In the eighth iteration, the ML-predicted top four sample configurations are perfectly consistent with the benchmark (FEM-calculated results). More importantly, our ML model is capable of recognizing all of the sample configurations with the best objective. 

Four kinds of indictors (MSE, MAE, EVS, R^2^) were monitored to evaluate the trained ML model within 10 iterations and are plotted in Figure 3b. Black curves indicate indicators for the training dataset, while red curves are for the testing dataset. In order to show the trends of the indicators with the increase in iteration numbers, the asymptote for indictors of the training dataset and the linear fit for indictors of the testing dataset are plotted by the black dashed lines and the red dashed lines, respectively. In the aspect of error-related indicators MSE and MAE, a lower value means a better trained ML model, with zero error corresponding to the value of 0. The asymptotic values for MSE and MAE of the training dataset are 5.71 × 10^−5^ and 5.68 × 10^−3^, respectively. The slopes of linear fit for MSE and MAE of the testing dataset are −1.31 × 10^−4^ and −1.94 × 10^−4^, respectively. With the increase in iteration numbers, compared to a continuously decreasing MSE for the testing dataset (from 1.55 × 10^−3^ in the 1st iteration to 3.17 × 10^−4^ in the 10th iteration), MSE for the training dataset decreases first and then fluctuates around the value as small as 5.17 × 10^−5^ after the seventh iteration. The same evolution trend is observed in MAE of the training and testing dataset. It can be concluded that at first, the ML model is highly biased with the input of a very small percentage of the entire design space (100 of 6435). Then, with more and more outliers being captured, corrected by FEM, and added to the input dataset, the ML model is informed to gradually mitigate the bias. The monotonically decreasing trends of MSE and MAE for the testing dataset with the increase in iteration numbers indicate a positive intervention. 

In the aspect of accuracy-related indictors EVS and R^2^, a higher value means a better trained ML model, with 100% accuracy corresponding to the value of 1. After the seventh iteration, EVS for the training dataset gradually leveled out at a value as high as 0.96, and EVS for the testing dataset continuously improved from −0.17 in the 1st iteration to 0.71 in the 10th iteration. The trend of R^2^ for both the training dataset and the testing dataset is the same as that of EVS. The EVS and R^2^ for the testing dataset exceed 0.6 after the eighth iteration. The monotonically increasing trends of EVS and R^2^ for the testing dataset with the increase in iteration numbers also verify the trained ML model becoming more and more accurate iteratively. By LSTM-based optimal strategy, the two best sample configurations (staggered structure) are obtained after only five iterations and maintained afterward. The top four best sample configurations are obtained after only eight iterations. Moreover, eight iterations with ~800 FEM-calculated samples as the input can train an eligible ML model not only for capturing all top four sample configurations efficiently but also for making a basic judgment to the objective of samples with any configurations. 

**Figure 4:**
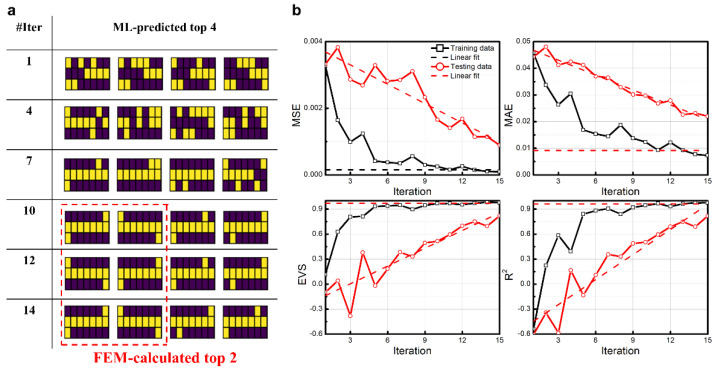
Iterated results and evaluations for the calculation domain discretized into 21 blocks by the proposed LSTM-based optimal strategy. (**a**) Top 4 ML-predicted sample configurations in each iteration. The sample configurations inside the red dashed-lined domain are the FEM-calculated top 2 configurations. (**b**) The relationship between evaluation indictors MSE, MAE, EVS, and R^2^ and iteration numbers. Black curves with open rectangles are for the training dataset; black dashed lines mark average values between iterations 11 and 15. Red curves with open circles are for the testing dataset; red dashed lines mark linear fit of red curves between iterations 1 and 15.

We further verify the generality and universality of the LSTM-based optimal strategy by considering the calculation domain discretized into 7 × 3 = 21 blocks. For 21 blocks, the entire design space includes ~10^6^ distinctive sample configurations. Figure 4 shows the corresponding iterated results and evaluations. The top two FEM-calculated sample configurations appear and are maintained as the ML-predicted top two after the 10th iteration, which is based on less than 1% of the entire design space. The MSE and MAE for the training dataset continuously decrease until the 10th iteration and stabilize at around 1.53 × 10^−4^ and 0.92 × 10^−2^ afterward. The EVS and R^2^ for the training dataset stabilize at around 0.97 and 0.96 after the 10th iteration. All four kinds of evaluations for the training dataset indicate that the iterated ML model for the training dataset has converged to a highly accurate version gradually. For the testing dataset, the MSE, MAE, EVS, and R^2^ of the ML model in the 15th iteration are 8.91 × 10^−4^, 2.20 × 10^−2^, 0.82, and 0.82, respectively. Moreover, the monotonically decreasing trends for MSE and MAE and the monotonically increasing trends for EVS and R^2^ would continue after the 15th iteration, which indicates that our ML model is not overfitted and still has space to improve.

## 4. Discussion

### 4.1. Comparisons with CNN-Based Optimal Strategy

**Figure 5:**
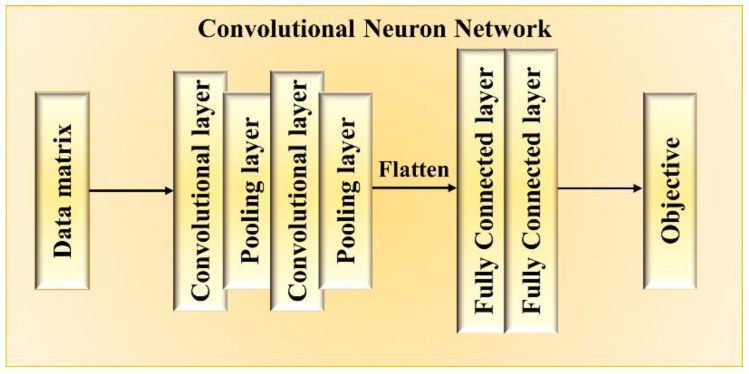
Construction of the convolutional neural network model.

Our optimal strategy is significantly dependent on the performance and robustness of LSTM. We further replaced the ML model in the flow chart (Figure 1) with CNN to explore the effect of the selection of different ML models. Similar to previous works [21,23], we deployed a conventional CNN as illustrated in Figure 5, which consists of two convolutional layers for feature learning and two fully connected layers for regression. Each convolutional layer outputs 32 filters by using a 3 by 3 kernel. The padding of “same” and the activation function of ReLU are adopted. Each convolutional layer is followed by a pooling layer with pool size of 2 by 2. The two followed fully connected layers are determined by the neuron number of 32, dropout ratio of 0.2, and ReLU activation function.

**Figure 6:**
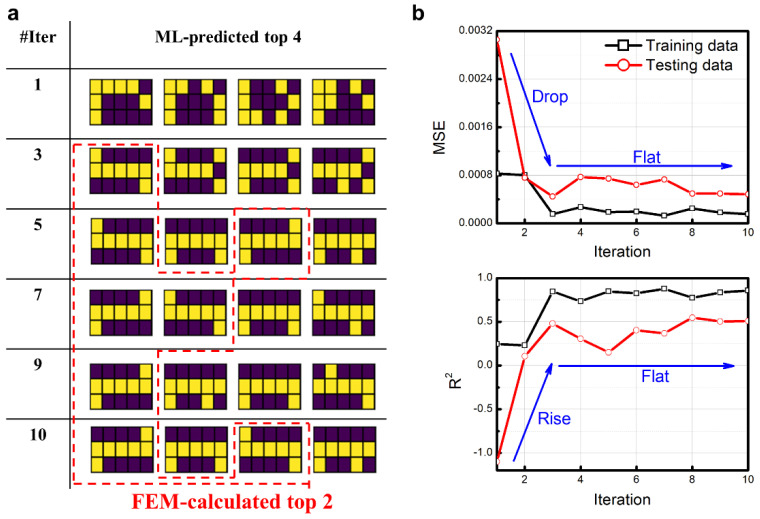
Iterated results and evaluations for the calculation domain discretized into 15 blocks by the CNN-based optimal strategy. (**a**) Top 4 ML-predicted sample configurations in each iteration. The sample configurations inside the red dashed-lined domain are the FEM-calculated top 2 configurations. (**b**) The relationship between evaluation functions MSE and R2 and iteration numbers. Black curves with open rectangles are for training dataset. Red curves with open circles are for testing dataset.

By the CNN-based optimal strategy, we revisited the problem of searching for sample configurations with the best objective for the calculation domain discretized into 15 blocks. Figure 6a shows the corresponding ML-predicted top four sample configurations in different iterations. The sample configurations inside the red dashed-lined domain are the FEM-calculated top two configurations. In the fifth iteration, the FEM-calculated top two sample configurations appear in the first and third places. In the seventh iteration, the ML-predicted top two configurations are consistent with the FEM-calculated top two configurations. However, in the ninth iteration, one of the FEM-calculated top two configurations disappears from the ML-predicted top four. In the 10th iteration, the FEM-calculated top two sample configurations appear in the first and third places. The shifting of the FEM-calculated top two sample configurations indicates that the CNN model is not becoming more and more accurate or stable with the increase in iteration numbers. Besides, the FEM-calculated top two sample configurations, which share the same objective, cannot be identified as apposable by the CNN model. The red dashed-lined domain in Figure 6a is not as stair-like as that in Figure 3a.

From the aforementioned analysis to the LSTM-based optimal strategy, one can see that MSE and MAE share the same trend, as do the EVS and R^2^. Therefore, here MSE and R^2^ were utilized to show the evolutions of error and accuracy of the CNN model. Figure 6b plots the relationship between EVS, R^2,^ and iteration numbers. The MSE for both the training dataset and the testing dataset drops before the third iteration but flattens out afterward, which indicates that the CNN model is basically converged after the third iteration, and the further increase in the iteration numbers could not make obvious improvements. In the 10th iteration, the MSE values for the training and testing datasets are 1.53 × 10^−4^ and 4.85 × 10^−4^, respectively, which are larger than those from the LSTM model in the 10th iteration. The R^2^ values for both the training dataset and testing dataset rise before the third iteration and flatten out afterward, sharing the same trend with the MSE. In the 10th iteration, the R^2^ values for the training dataset and the testing dataset are 0.86 and 0.51, respectively, indicating the inadequacy compared with the LSTM model. Considering the intrinsic logic of CNN and LSTM, it can be concluded that in material design and structural optimal fields, if the material ratio is fixed and the specific property is cared about, LSTM may be a better approach.

### 4.2. Failure Mechanism of the Optimal Staggered Structure

**Figure 7:**
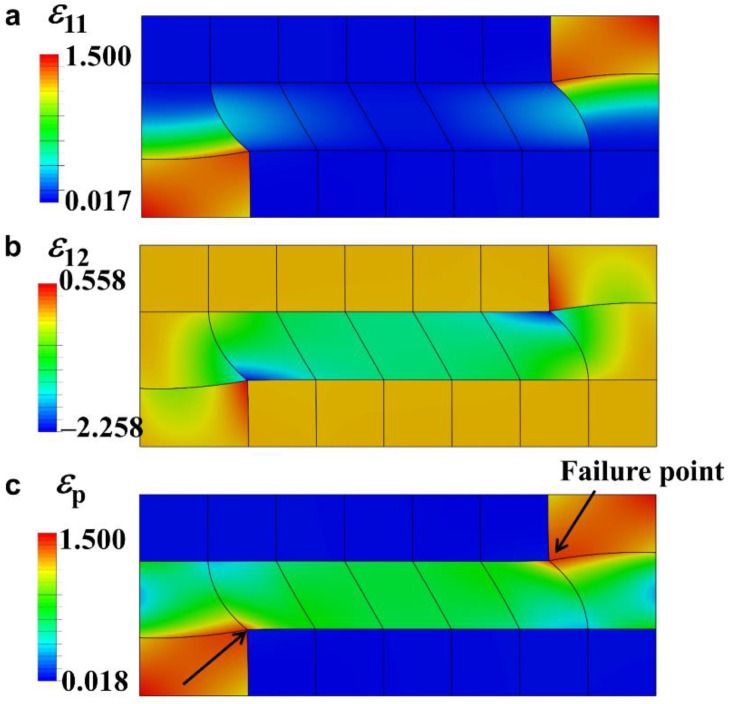
The distribution of (**a**) tension strain *ε*_11_, (**b**) shear strain *ε*_12_, and (**c**) maximum principal strain *ε*_p_ of the optimal staggered structure at the moment of failure.

From the aforementioned analysis, our LSTM-based optimal strategy confirms the global optimality of the staggered structure in the aspect of simultaneous superior stiffness and toughness. For the concrete deformation behavior of the staggered structure, Figure 7 plots the distribution of tension strain, shear strain, and maximum principal strain at the moment of failure. Under the failure criterion of maximum principal strain, one can see that the soft block located in the vicinity of the corner of the hard platelets fails first. However, when this soft block reaches failure, other blocks can still bear load, which means that not all materials are utilized to 100%. If the material properties of the soft blocks can be further adjusted to accommodate the potential failure point, the sample may be able to bear more loading. With the rapid development of additive manufacturing and machine learning, we speculate that if we could provide more flexible options for the evolution, the optimal sample configuration could be adjusted to bear larger failure strain in the corner while relatively lower failure strain in other parts, and then the artificial staggered structure may be stronger than natural materials.

## 5. Conclusions

We have developed a novel LSTM-based strategy to search for the optimal design of bio-inspired composite materials with simultaneously superior stiffness and toughness at the nanoscale level. The LSTM-based optimal strategy is applicable to a system with multiple best sample configurations, verified by the calculation domain discretized into 15 and 21 blocks. In all cases, the staggered structure is proved to be globally optimal in the aspect of simultaneous superior stiffness and toughness. Our LSTM-based optimal strategy has three main advantages: (1) It can generate an ML model with accuracy greater than 0.6 for the testing dataset based on less than 10% of the entire design space, and the increasing trend of the accuracy indicators continues with the increase in iteration numbers. (2) It can obtain globally optimal sample configurations within only 5 and 10 iterations for calculation domains discretized into 15 and 21 blocks, respectively. (3) It can capture multiple sample configurations with the best objective. In the future, our LSTM-based optimal strategy could be combined with additive manufacturing to further improve the performance of the staggered structure by customizing the material properties. Our LSTM-based optimal strategy also has the potential to be applied to many similar material design problems with multiple optimal distribution patterns under the prerequisite of the conservation of mass.

## Data Availability

Data that support the findings of this study are available from the corresponding author upon reasonable request.
